# 

**Published:** 1895-10

**Authors:** 


### CONTENTS.

ORIGINAL ARTICLES—

A Contribution to the Study of the Ætiology, Symptomatology and Treatment of Endometritis. By George Tucker Harrison, M. A., M. D., New York .......... 495

A Case of Pemphigus. By A. H. Ohmann-Dumesnil, M. D., St. Louis .......... 508

Hemorrhoids; Prolapsed Rectum; New Operation. By Merrill Ricketts, M. D., Cincinnati .......... 512

The General Therapeutic Effect of the Alternative Electric Current of High Frequency and of High Tension. By Dr. G. Apostoli, of Paris .......... 514

Electrotherapy as a Means of Diagnosis in Gynæcology. By Dr. G. Apostoli, of Paris .......... 517

SOCIETY REPORTS—

Proceedings of the Virginia Medical Society .................... 520

SELECTIONS AND ABSTRACTS—

The Treatment of Intermittent Fever. By Walter M. Flemming, M. D., New York City .................... 533

On the Administration of the Salicylates in Acute Rheumatism 536

Editorial .................... 539
Notes .................... 541
Special Notes .................... 544
Prescriptions .................... 548–549

